# Simultaneous Left Ventricular and Deep Vein Thrombi Caused by Protein C Deficiency

**DOI:** 10.1155/2017/4240959

**Published:** 2017-01-17

**Authors:** Harufumi Maki, Motohiro Nishiyama, Motoaki Shirakawa

**Affiliations:** Department of Surgery, Fujieda Municipal General Hospital, 4-1-11 Surugadai, Fujieda, Shizuoka 426-8677, Japan

## Abstract

Protein C deficiency is a risk of venous thrombosis because of poor fibrinolytic activity. It remains controversial whether protein C deficiency causes arterial thrombosis. A 21-year-old woman was referred with a chief complaint of right leg pain and numbness. Contrast-enhanced computed tomography revealed a low-density mass in the left ventricle (LV), splenic infarction, and peripheral arterial obstructions in her right leg. Thrombosis extending from the renal vein to the inferior vena cava was also detected. Electrocardiography revealed ST depression in leads II, III, and aVF. Transthoracic echocardiography revealed hypokinesis of the apex and interventricular septum and a hypoechoic mass in the LV (26 × 20 mm). She was diagnosed with acute arterial obstruction caused by the LV thrombus, which might have resulted from previous myocardial infarction. Protein C activation turned out to be low (41%) 5 days after admission. The anticoagulant therapy was switched from heparin to rivaroxaban 16 days after admission. The LV thrombus disappeared 24 days after initial treatment, and she has had no thrombotic episodes for 2.8 years under rivaroxaban therapy. Thrombophilia should be investigated for cases of simultaneous left ventricular and deep venous thrombi. Rivaroxaban can be effective in prevention of further thrombotic events.

## 1. Introduction

Protein C deficiency occurs in one of every 200–500 people [1], and patients with protein C deficiency have a 7-fold higher risk of developing thrombosis than healthy people because of poor fibrinolytic activity. Protein C deficiency results mainly in thrombosis of the pulmonary veins, deep veins of the extremities, and mesenteric veins [2]. It remains controversial whether protein C deficiency causes acute arterial occlusion such as myocardial infarction, cerebral infarction, and peripheral arterial occlusion [3–6]. Novel oral anticoagulants (NOAC), which directly inhibit thrombin or factor Xa, are a possible alternative therapeutic option to warfarin, a vitamin K antagonist, to prevent clot formation [7, 8].

Left ventricular (LV) thrombosis was present in 7–46% of patients after acute myocardial infarction and thromboembolic complications occurred in 2-3% of patients with LV thrombosis [9]. Warfarin therapy is the standard of care for the treatment of LV thrombosis [9], with only a small number of case reports evaluating the efficacy of NOACs such as dabigatran and rivaroxaban [10–12].

We presented herein a patient of simultaneous left ventricular and deep vein thrombi with protein C deficiency. We struggled for appropriate anticoagulant therapy.

## 2. Case Presentation

**Figure 1:**
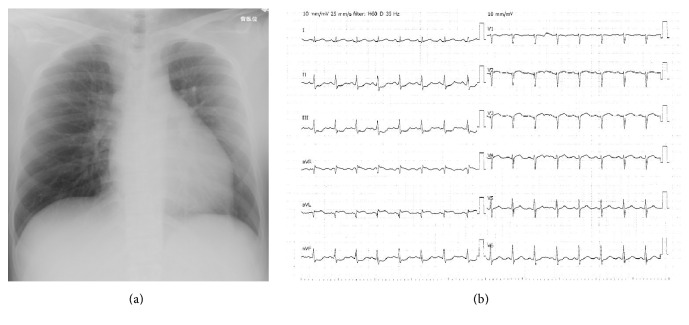
(a) Chest X-ray showing cardiomegaly. (b) Electrocardiogram showing tachycardia. ST-T depression was seen in leads II, III, and aVF.

**Figure 2:**
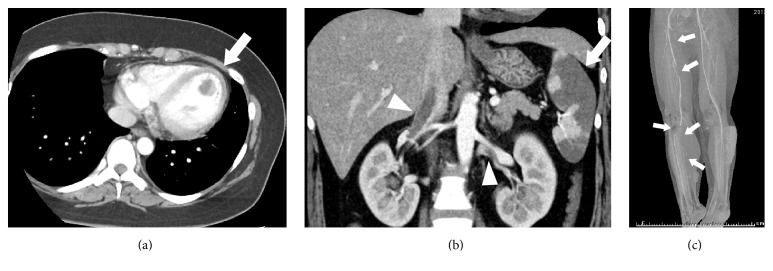
CT findings. (a) A ball-like thrombus located in the left ventricle (arrow). (b) Splenic infarction (arrow) and thrombi in bilateral renal veins and the inferior vena cava (arrowheads). (c) 3D reconstruction image of the lower limbs showing defects in the right superficial femoral artery, right deep femoral artery, right popliteal artery, right posterior tibial artery, and right peroneal artery (arrows).

**Figure 3:**
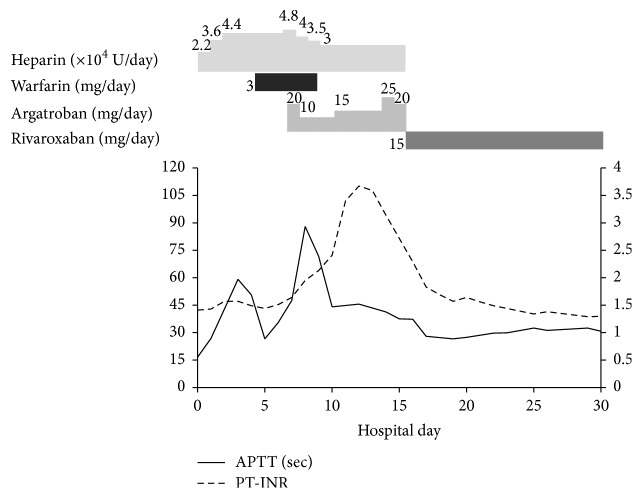
Trends in anticoagulant therapy. APTT: activated partial thromboplastin time; PT-INR: prothrombin time expressed as international normalized ratio.

A 21-year-old woman was referred to our hospital with a chief complaint of right leg pain and numbness. Cold sensation, pallor, and foot-drop of her right lower extremity were observed on admission. She had no medical history related to thrombosis except for obesity. She was 161 cm tall and weighed 86 kg, with a body mass index of 33.3 kg/m^2^. Activated partial thromboplastin time (APTT) was 16.6 s, prothrombin time (expressed as international normalized ratio, PT-INR) 1.41, D-dimer: 1.8 *μ*g/mL, and antithrombin III level 86%. Chest X-ray revealed a cardiothoracic ratio of 52% (Figure 1(a)), and electrocardiography revealed sinus tachycardia and ST depression in leads II, III, and aVF (Figure 1(b)). Contrast-enhanced computed tomography revealed a low-density mass in the LV (Figure 2(a)), splenic infarction (Figure 2(b)), and peripheral arterial obstructions of her right leg (Figure 2(c)). Thrombosis extending from the renal vein to the inferior vena cava was also detected (Figure 2(b)). Transthoracic echocardiography revealed hypokinesis of the apex and the interventricular septum and a hypoechoic mass with pendulum-like motion in the LV (26 × 20 mm). She had no patent foramen ovale. She was diagnosed with acute arterial obstruction caused by the LV thrombus, which may have resulted from previous myocardial infarction. Heparin therapy was chosen over thrombectomy for the right lower extremity or the LV thrombi. After bolus administration of 3000 U of heparin, continuous infusion of 22,000 U/day of heparin was started. APTT remained within the normal range despite increasing heparin to 44,000 U/day 4 days after hospitalization. 5 days after hospitalization, protein C activation on admission turned out to be low (41%), protein S activation was 89%, lupus anticoagulant was 0.91, and anticardiolipin antibody was 8.0 U/mL. Arterial obstruction of her left leg occurred 6 days after admission. Warfarin therapy was added because her left leg was not paralyzed and antithrombin III level had decreased to 45% at this time. Argatroban was added 8 days after admission because APTT remained below the therapeutic range and warfarin was stopped because of PT-INR prolongation. We changed to 15 mg/day of rivaroxaban 16 days after admission because the APTT normalized again and new thrombi had developed in both legs. The LV thrombus disappeared 24 days after initial treatment. Her treatment course is summarized in Figure 3. Right below-knee amputation was performed 30 days after admission for pain control and rehabilitation. The postoperative course was uneventful, and she has had neither repeat thrombosis nor bleeding episodes for 2.8 years under anticoagulation therapy with the same dose of rivaroxaban.

## 3. Discussion

We present a patient of simultaneous acute arterial obstruction caused by an LV thrombus and deep vein thrombosis with protein C deficiency. Thrombophilia should be investigated for cases of simultaneous left ventricular and deep venous thrombi. For the treatment, administration of rivaroxaban can contribute to prevention of further thrombotic events.

In our patient, peripheral arterial obstructions resulted from the LV thrombus because of myocardial infarction, which was diagnosed based on cardiac ultrasonographic findings and electrocardiographic changes although coronary angiography was not performed. LV thrombosis can be caused by LV dysfunction, an ejection fraction < 35%, anterior infarction, apical dyskinesis, or aneurysm, whereas it is rarely caused by decreased activity of both protein C and protein S [13]. However, some thrombophilia was suspected in our patient because of deep vein thrombi simultaneously detected by computed tomography. Protein C deficiency should be considered as the cause of simultaneous LV thrombosis and deep vein thrombosis, especially in patients without major risk factors for coronary artery disease. General anesthesia was thought to carry a high risk of further embolic events in our patient; however emergent thrombectomy under local anesthesia could be attempted for limb salvage.

It was difficult to select appropriate coagulation therapy in our patient. Warfarin therapy is recommended for LV thrombosis after myocardial infarction rather than surgical removal according to both the European Society of Cardiology and the American Heart Association guidelines [9]. Recently, Nagamoto et al. reported resolution of an LV thrombus resulting from previous myocardial infarction 27 days after dabigatran initiation [10]. Chung et al. also reported resolution of an LV thrombus with acute ischemic stroke and atrial fibrillation 7 days after dabigatran initiation [11]. Nakasuka et al. reported resolution of an LV thrombus secondary to tachycardia-induced heart failure 7 days after rivaroxaban initiation [12]. Further studies are needed to confirm the efficacy of NOAC for treating left ventricular thrombosis.

Moreover, it was difficult to control APTT values because our patient showed heparin resistance. Heparin resistance is defined when APTT values do not increase 1.5 times following the administration of more than 35,000 units of heparin per day [14, 15]. Heparin-induced thrombocytopenia was ruled out in our patient because of the absence of thrombocytopenia. Argatroban was temporarily useful; however APTT values again decreased to the normal range. Argatroban resistance has been reported rarely. Therefore, the mechanism of action and treatment are unknown [16]. Rivaroxaban is approved in the European Union and the United States to treat deep vein thrombosis and for the secondary prevention of recurrent venous thromboembolism [17]. Rivaroxaban could have potential efficacy for the treatment of simultaneous LV and deep vein thrombi with protein C deficiency like our patient.

## 4. Conclusions

Thrombophilia should be investigated for cases of simultaneous left ventricular and deep venous thrombi. Rivaroxaban can have an effect on prevention of further thrombotic events.

## Abbreviations

LV: Left ventricle
NOAC: Novel oral anticoagulants
APTT: Activated partial thromboplastin time
PT-INR: International normalized ratio of prothrombin time.

## Data Access

Data sharing not applicable to this article as no datasets were generated or analysed during the current study.
